# The Assessment of Endovascular Therapies in Ischemic Stroke: Management, Problems and Future Approaches

**DOI:** 10.3390/jcm11071864

**Published:** 2022-03-28

**Authors:** Tadeusz J. Popiela, Wirginia Krzyściak, Fabio Pilato, Anna Ligęzka, Beata Bystrowska, Karolina Bukowska-Strakova, Paweł Brzegowy, Karthik Muthusamy, Tamas Kozicz

**Affiliations:** 1 Department of Radiology, Medical College, Jagiellonian University, 31-501 Kraków, Poland; p.brzegowy@uj.edu.pl; 2 Department of Medical Diagnostics, Medical College, Jagiellonian University, 31-688 Kraków, Poland; ligezka.anna@mayo.edu; 3 Neurology, Neurophysiology and Neurobiology Unit, Department of Medicine, Università Campus Bio-Medico di Roma, 00128 Rome, Italy; f.pilato@policlinicocampus.it; 4 Department of Clinical Genomics, Mayo Clinic, Rochester, MN 55902, USA; muthusamy.karthik@mayo.edu (K.M.); kozicz.tamas@mayo.edu (T.K.); 5 Center for Individualized Medicine, Mayo Clinic, Rochester, MN 55902, USA; 6 Department of Toxicology, Medical College, Jagiellonian University, Medyczna 9, 30-688 Kraków, Poland; beata.bystrowska@uj.edu.pl; 7 Department of Clinical Immunology and Transplantology, Institute of Pediatrics, Medical College, Jagiellonian University, 30-663 Kraków, Poland; k.bukowska-strakova@uj.edu.pl

**Keywords:** ischemic stroke, thrombolysis, rt-PA, endovascular therapies, mechanical thrombectomy, clots, mitochondria

## Abstract

Ischemic stroke accounts for over 80% of all strokes and is one of the leading causes of mortality and permanent disability worldwide. Intravenous administration of recombinant tissue plasminogen activator (rt-PA) is an approved treatment strategy for acute ischemic stroke of large arteries within 4.5 h of onset, and mechanical thrombectomy can be used for large arteries occlusion up to 24 h after onset. Improving diagnostic work up for acute treatment, reducing onset-to-needle time and urgent radiological access angiographic CT images (angioCT) and Magnetic Resonance Imaging (MRI) are real problems for many healthcare systems, which limits the number of patients with good prognosis in real world compared to the results of randomized controlled trials. The applied endovascular procedures demonstrated high efficacy, but some cellular mechanisms, following reperfusion, are still unknown. Changes in the morphology and function of mitochondria associated with reperfusion and ischemia-reperfusion neuronal death are still understudied research fields. Moreover, future research is needed to elucidate the relationship between continuously refined imaging techniques and the variable structure or physical properties of the clot along with vascular permeability and the pleiotropism of ischemic reperfusion lesions in the penumbra, in order to define targeted preventive procedures promoting long-term health benefits.

## 1. Introduction

Ischemic stroke is caused by interruption of the blood supply, most often by a blood clot [1]. The histological structure of the thrombus may determine the subtype of stroke, depending on its etiology: cardioembolic, atherothrombotic, or stroke of unknown cause [2], and it may influence reperfusion outcome. Fibrin and platelets dominate in cardioembolic thrombi with a small amount of neutrophil extracellular traps (NETs) which impair tissue plasminogen activator tPA-mediated thrombolysis [3]. In atherothrombosis, thrombosis, red blood cells and fibrin predominate, while the relative proportion of each component is undefined for cryptogenic strokes [4]. The knowledge of clot histological structure, percentage composition and architecture of the clot components, as well as the patient’s clinical features, and the molecular changes in the area of cerebral ischemia seem to be crucial for understanding the results of the current acute stroke treatments and long-term clinical benefits of patients with ischemic stroke [5,6,7].

Ischemic stroke annually affects 17 million people worldwide, of which as many as 6 million die, and a significant proportion are permanently burdened with disability [8,9,10]. Ischemic stroke accounts for approximately 80% of all stroke cases (0.2% according to Béjot et al. refers to the world population and applies to all stroke cases in 2016; 0.2% is estimated as 7.9 billion people lived in the world in 2021) [11,12,13]. Estimates indicate that the increase in stroke incidence in the general population will reach 25% between 2010 and 2030; in men aged 65 to 74, it accounts for approximately 70 cases per 100,000, while in the general population over 74, women are more likely to experience acute stroke with more severe consequences [14,15]. The projected number of strokes in all EU countries, Iceland, Norway, and Switzerland, will increase by 27% (from 1.1 million in 2000 to 1.5 million in 2025) [16,17,18,19].

Studying data collected by Global Burden of Disease, Injuries, and Risk Factors Study 2017 (GBD 2017), including separate estimates of the global burden and trends for each type of stroke, a 2-fold increase was observed in the absolute number of people who had a new stroke, died, survived, or remained disabled after their stroke within the 12 million stroke-related incidents. Most of the stroke burden occurred in low- and middle-income countries, 65% of those stroke cases were ischemic stroke, 26% was primary intracerebral haemorrhage (PICH), and 9% were represented by subarachnoid haemorrhages [20,21,22].

The above-mentioned epidemiological data related to the incidence of stroke varies from country to country and fluctuates according to the burden in high-, low-, and mid-dle-income countries. Individual records from hospital stroke units are incomplete and their availability is limited [23,24]. The increase in the number of registered cases of people suffering from ischemic stroke will be found mainly in the populations of developing countries [14,25].

One of the reasons for the increase in morbidity in the world is the change in the structure of the population—the high level of aging compared to the number of births. Moreover, in the last two years, COVID-19 pandemic deeply affected stroke management [15] and, probably, even more effects will be evident in the future, especially in younger people [16,17,18,19]. Based on the meta-analysis of patients with COVID-19 developing acute cerebrovascular diseases, it was observed that among the elderly, stroke episodes occurred more frequently in patients with comorbidities, i.e., diabetes, coronary artery disease, and severe infections, as compared to patients who had a stroke without infection. Younger people with registered stroke cases had higher NIHSS, higher incidence of large vessel obstruction, and higher in-hospital mortality [26]. It may be due to the inflammatory response in endothelial cells, characterized by increased cytokine secretion and expression of adhesion molecules [27].

In Poland, in 2050, it has been estimated that the society will consist of 11% of people in the pre-working age, 57% in working age, and 32.7% over 65 years [28]. Ischemic stroke is associated with almost twice as many cases of dementia compared to the general population. The dramatic increase in the incidence of stroke in the population prompts epidemiologists to describe it as a form of a pandemic [29]. This together means that all efforts to improve epidemiological data take a high priority of healthcare systems, both in terms of primary prevention of stroke occurrence and secondary prevention of subsequent episodes [30]. Stroke is not only one of the most common causes of hospitalization, but also comes at a high economic cost. Moreover, stroke is a main problem not only in older ages but also in young adult causing increased long-lasting health-related costs [31]. Today, about 34% of total healthcare expenditure in the world is spent on this disease. The reason for the increased economic burden of stroke treatment is the need for long hospitalization and rehabilitation services, which are the basis of conservative treatment [32].

## 2. Qualification for the Causal Treatment of Ischemic Stroke

Currently approved treatments for acute ischemic stroke (AIS) are mechanical thrombectomy (MT), which accounted for 1.9% of treatments in 2019 in Poland (TM was performed in 1413 patients out of 75,213 of all patients hospitalized due to ischemic stroke) and 3.5% of treatments in 2020 year (TM was performed in 2526 patients out of 70,926 of all patients hospitalized due to ischemic stroke) [33] while in the United States, MTs was performed (3.1% out of examined acute ischemic stroke population, i.e., approximately 424,330; data from 2016) [22]. On the other hand, the intravenous thrombolysis (IVT) with the administration of the recombinant tissue plasminogen activator (rt-PA) accounts for 7.3% treatments of AIS patients in Europe [34,35] and 3.6–6.5% treatments of AIS patients in the USA [36,37,38].

Current stroke guidelines report that a fast diagnostic protocol, in qualifying stroke patients for acute treatments should be performed [39]. First step is the exclusion of a hemorrhagic stroke. For this purpose, a computed tomography (CT) of the head or a magnetic resonance imaging (MRI) is performed. Due to the limited availability of the latter, a CT of the head is more used and faster than MRI in acute setting. After the exclusion of cerebral hemorrhage and the features of a completed ischemic stroke in the CT scan, intravenous thrombolytic therapy is started with the administration of rt-PA within a 4.5 h time window from the onset of the first clinical symptoms of ischemic stroke. When the stroke etiology is a large vessel occlusion (LVO) in anterior circulation, MT is the standard of care for acute stroke [39]. It should be noted that in patients with an anterior cerebral ischemic stroke, the results of MT started within 4.5 h alone compared with the combined IVT, measured on day 90 on the modified Rankin scale (mRS) were comparable. These were the results of a multicenter randomized non-inferiority clinical trial conducted at 33 stroke centers in China. Only the clinical evaluation related to functional improvement after 90 days (0–2 in mRS) was taken into account as the endpoint [40]. Moreover, MT is the treatment of choice when rt-PA is contraindicated, i.e., in patients treated with anticoagulants. Depending on time of stroke onset, the qualification is based on additional specialist examinations, such as CT angiography (angioCT) and CT perfusion (pCT). In the case of occlusion of large vessels in the anterior cerebral circulation, the DAWN and DEFUSE criteria apply in patients within 6 to 24 h after symptom onset [41,42] and depending on availability, the CT scan may be replaced with appropriate MR sequences.

### 2.1. A Standard Operating Procedure of Imaging Methods Which Is Employed in Ischemic Stroke in NSSU Diagnostic Imaging Unit

A 56-year-old patient with symptoms of ischemic stroke of the right hemisphere which appeared 2 h before admission to the hospital emergency department was demonstrated.

Two years ago, the patient had his first ischemic stroke of the left hemisphere of the brain, which left him with only slight neurological deficits—mRS 1. At the present admission to the hospital, he was confused and had full left limb paresis—NIHSS 16. The CT scan was started 2 h 20 min after the onset of the symptoms of the stroke. The film (https://drive.google.com/file/d/1xRIdjTL7EYZZz0cSCqWGEr49Y2AbPnj9/view?usp=sharing, accessed on 27 February 2022) presents the imaging techniques used in sequence:

1. CT scan without contrast assisted by RAPID software. The scans automatically analyzed (RAPID software) on the ASPECT scale show only old ischemic changes in the left hemisphere of the brain. In the right hemisphere of the brain there are no signs of the presence of hypodense areas that would indicate new areas of stroke.
2. Perfusion CT scan assisted by RAPID software. The reference levels in the RAPID analysis are set according to the criteria developed in the DEFUSE 3 study. The levels of 2 parameters are investigated: CBF (cerebral blood flow) less than 30% compared to the opposite hemisphere indicates necrosis—purple color area, Tmax value greater than 6 sec signifies the area of the penumbra. Additionally, a quantitative analysis is performed. The calculated mismatch is at the level of 1.6, which according to the adopted DEFUSE 3 criteria, should disqualify the patient from mechanical thrombectomy. On the other hand, European recommendations suggest that up to 6 h after the appearance of the first symptoms of the disease, we do not always have to follow the pCT results, but rather rely on the CT scan.
3. CT angiography of the cerebral and intracerebral arteries taken from the level of the aortic arch. The patient moved during the examination, because of this, the head is bent to the right and slightly upwards. Full occlusion of the right internal carotid artery is visible in its proximal part.
4. MR Diffusion Weighted Imaging (DWI) and corresponding apparent diffusion coefficient (ADC) sequences. A. In the DWI sequence a large area with a hyperintense signal corresponding to cerebral ischemia is visible in the right hemisphere. B. In the ADC sequence the same area has a hypointense signal which may correspond, including DWI images, to the acute nature of ischemia.
5. MR Fluid-attenuated inversion recovery (FLAIR) and DWI sequences. DWI-FLAIR-mismatch. No marked parenchymal hyperintensity is detected on fluid attenuated inversion recovery (FLAIR) images on right hemisphere (C), while acute ischemic lesion is clearly visible on DWI (D), indicating DWI-FLAIR-mismatch. Old post-stroke lesions are visible in the left hemisphere of the brain.
6. Digital Subtraction Angiography—DSA. The distal end of the aspiration catheter through which a contrast agent is administered is inserted into the proximal segment of the right internal carotid artery. The place of the occlusion is visible through which the contrast agent does not flow.
7. Rotary Digital Subtraction Angiography—3D DSA. Control examination performed directly after successful mechanical thrombectomy—mTICI 3. Visible flow of contrast blood both through the main trunk of the internal carotid artery and its branches.

### 2.2. Prehospital Triage in Ischemic Stroke: Problems and Needs in Poland

Substantial uncertainty exists on the benefit of organizational paradigms in stroke networks and several studies compared functional outcome between the mothership and the drip and ship models [43] and probably they mainly depend on local infrastructures and organization.

As a result of the VII Symposium on Acute Brain Stroke and the experiences of Polish leaders of the Pilot Mechanical Thrombectomy in Poland on 2–4 December 2021 [44], attention was drawn to the difficulties associated with the already applied endovascular treatment in ischemic stroke in Poland, i.e., difficult fast transport (from call to realization) for endovascular treatment (EVT) of patients with acute ischemic stroke due to intracranial large vessel occlusion (LVO) with appropriate final qualification for causal treatment (rt-PA and TM) and access to urgent brain scan (angioCT and MRI). The criteria for qualifying for the performance of MT (including, e.g., angioCT) in coordinated care due to ischemic stroke are a real problem that can be solved systematically by introducing, inter alia, training for teams of neuro-interventionalists in causal treatment. One of the elements of the training would be the detection of LVO, i.e., middle cerebral artery (M1), the proximal second segment of the middle cerebral artery (M2), and the internal carotid artery (ICA) in CT, preceded by cross-validation on pre-processed images (which are the standard in image learning methods) as support for neurointervention centers of acute stroke that do not have radiologists on-site. This is based on unsupervised analysis of convolutional neural networks (CNNs) for the detection of LVO in angioCT (used in training in Nashville, TN, USA) [45]. The presented approach would allow for the elimination of costs related to unjustified transport to the superior intervention center, guaranteeing the continuity of the hospital’s operation.

Another problem is the identification of high-risk patients (with intracranial occlusion) on the basis of the observed clinical symptoms or individual assessment of the prognosis after endovascular procedures.

Creating a pilot network with referring patients to a superior center (depending on the occlusion of large vessels detected in angioCT) as a reference center would allow avoiding local problems related to a shortage of specialist local staff, and facilitate a personalized approach, guaranteeing the comfort of work of all persons participating in the pilot project as it was done in Poland, like as in China [46].

Incomplete formalized reimbursement services for the mechanical thrombectomy (related to the lack of dedicated procedures) are also a real problem, which, in the case of randomized trials, show that the use of EVT as an option for the treatment of acute ischemic stroke eliminates the costs associated with patient care during the first year of stroke and generates savings in the next years of the patient’s life [47,48]. In the case of staffing problems and the growing number of stroke patients due to the aging society, the solution could be employment standards analogous to those in intensive care units: 1:1 or 1:2.

The presented problems related to delays in EVT concern many healthcare systems [48], thus limiting the number of patients with a good prognosis compared to the results of randomized trials.

In a randomized clinical trial to evaluate rapid EVT of ischemic stroke, quality control was highlighted as a guideline for ultimate success in the treatment of causative agents with weekly monitoring of imaging speed with feedback to central centers via teleconference [49]. Training in fast and effective EVT and imaging methods has helped meet these requirements. The time criterion was met with a validated time from non-contrast CT scan to groin puncture of up to 60 min and from non-contrast CT scan to first reperfusion (with first median cerebral artery flow) of <90 min. Meeting the strict time criteria goals ensured the introduction of an effective EVT with much better prognosis for patients with acute ischemic stroke. A clear limitation in the use of endovascular procedures was: difficult accessibility due to the tortuosity of the cerebral vessels or the unavailability of the neuro-interventional team, which coincides with the problems of the non-randomized studies presented above.

Ongoing efforts should aim to precisely define the occurrence of intracranial occlusion (LVO) in the transmitted available imaging test results (angioCT) with a precise estimation of the time needed to influence pre-hospital triage decisions in order to eliminate delays in causal treatment of individual patients. Successive efforts should focus on identifying real obstacles and problems guaranteeing the improvement of pre-hospital triage and the availability of pre- and post-processing IT systems, combined with timely monitoring of causal treatment of ischemic stroke and long-term care.

Future efforts should also counter the importance of telemedicine facilitating the pre-registration process using devices with GPS navigation applications, which will further facilitate the proper selection of data based on real-time information with the possibility of verifying times: while driving with synchronization of the work time of the neuro-intervention team that will review current regimens and provide long-term health benefits in patient care for ischemic stroke induced by intracranial LVO.

## 3. Causes of Reperfusion Procedures Failure and Potentials Risks Related to Reperfusion

Intravenous thrombolysis with rt-PA and endovascular thrombectomy are the current standard of care for acute ischemic stroke [50,51]. Their beneficial effect is mainly due to reperfusion of cerebral vessels occlusion. However, there is a proportion of patients who do not achieve clinical improvement despite successful recanalization of the occluded artery and reperfusion of the ischemic area. Despite successful recanalization by endovascular procedures, some neurological impairments may be unrecovered and some patients may develop early complications of ischemic stroke, including early neurological deterioration, symptomatic hemorrhagic transformation (so-called secondary hemorrhage) and cerebral edema [52,53,54], but the causes of reperfusion injuries are still debated.

The pathophysiology of reperfusion injuries include: damage to the blood–brain barrier (BBB) [55], structural remodeling of endothelial cells associated with breaking tight connections, a consistently non-specific inflammatory response induced by oxidative, nitrosative (overproduction of reactive oxygen and nitrogen species) [56], and metabolic stress (associated with insufficient oxygen supply and hypoglycemia in neuronal mitochondria). In the event of reduced blood flow to the brain, apart from structural degeneration, there is also a functional dysfunction of blood vessels based on reduced Na^+^/K^+^-ATPase activity, overload of cells with sodium, calcium [57], partial depolarization, and loss of membrane potential, resulting in an increased inflow of sodium ions. This, in turn, leads to the penetration of chlorine ions and water inside the cell, causing swelling of neurons [58,59]. Subsequently, a cascade of biochemical processes related to the release of the main excitatory neurotransmitters, i.e., glutamic acid, produced from presynaptic terminals by depolarization of synaptosomes and hindered uptake by hypoxic astrocytes takes place [60]. Ischemia-induced (with blood flow to the brain below 20 mL/100 g/min), persistently elevated glutamate levels occur in all regions of the brain, leading to neurological activation associated with glutamate excitotoxicity in ischemic neurons [61]. As a consequence, continuous glutamatergic stimulation impairs the basic functions of mitochondria [62,63] and damages the BBB. It comes to an increased concentration of lactates, carbonate and glutamate, and a decreased level of alanine, citrate, glycine, tyrosine, methionine, and tryptophan in the peripheral blood [64], which emphasizes the key role of these amino acids in bio-energetic homeostasis in both ischemic brain areas and in the peripheral blood. Tyrosine, lactate, and tryptophan have been identified as potential biomarkers of acute ischemic stroke, which are associated with enhanced glycolysis and inhibition of the tricarboxylic acid cycle (TAC) in patients with acute ischemic stroke (AIS). Lactate as an indicator of the severity of anaerobic metabolism associated with ischemia and subsequent hypoxia is elevated in the cerebrospinal fluid, brain tissues, and blood serum of AIS patients. Additionally, the concentration of lactate reflects the level of neuronal necrosis and the prognosis after ischemic stroke [65].

Single results of clinical data in humans are inconclusive due to the occurrence of perifocal edema and leakage of the BBB by labeled metabolites, which complicate the interpretation of effectiveness of reperfusion procedures [66,67]. In addition, progressive neurocognitive disorders in more than half of stroke patients are still a severe and non-decreasing burden on healthcare systems around the world [68,69,70].

The effectiveness of the reperfusion procedures used in ischemic stroke may be limited by the secondary ischemia-reperfusion injury of the brain, which may increase the volume of ischemia and aggravate the cerebral infarction [71,72,73]. The results of both animal and human clinical trials show that despite early reperfusion after stroke, which protects healthy brain tissue from further ischemia, selective loss of peri-infarction neurons or emerging micro infarcts may be adverse effects resulting in long-term sensorimotor deficits after stroke [74]. An additional obstacle in the effectiveness of the performed reperfusion procedures is the lack of actual evaluation of the histopathological structure of the embolic material in real time or thrombus perviousness evaluated by neuroradiological techniques, which means that mechanical thrombus removal during thrombectomy may be quite difficult and not bring the expected results [75]. This is due to the fact that some clots require multiple removal attempts in order to achieve successful recanalization (with the preferred recanalization rate, classification of modified treatment in cerebral ischemia, mTICI), while other thrombi are effectively removed in the first attempt, resulting in better final results [76]. There are also situations where, despite a preliminary assessment of the seemingly known consistency of the clot, it turns out that the structure is so diverse that it may cause unexpected fragmentation of the clot during mechanical thrombectomy with its dispersion to the distal branches of the cerebral arteries extending the infarct zone [77]. The risk of clot defragmentation strictly depends on its composition, namely the dissection resistance increases significantly with an increase in the content of fibrin or neutrophilic networks (NETs, consisting of: DNA, histones, and proteolytic enzymes produced by activated neutrophils in various mechanisms) [78], while red blood clots are more prone to rupture during emerging stresses [79] but it also may depend on the patient’s features, i.e., antithrombotic therapy [7]. In the presence of NETs and related extracellular DNA and histones, the structure of fibrin changes, becoming more resistant to both mechanical destruction using EVT devices, thus increasing the difficulty of effectively removing the thrombus during mechanical thrombectomy and enzymatic degradation using rt-PA. Knowledge of the content and amount of NETs in the composition of the embolic material could, therefore, be a potential goal of assessing the effectiveness of mechanical thrombectomy in terms of successful recanalization [80]. This approach supports the concept of multi-target combined therapy (apart from the components, targeted to the percentage and architecture of the individual components of the clot structure) in the causal treatment of ischemic stroke of large vessels of the cerebral arteries [81].

With regard to the reperfusion procedures, the current efforts are focused on achieving complete reperfusion already during the first thrombectomy attempt as a favorable end result of EVT using mechanical thrombectomy and/or a strategy combining thrombectomy with thromboaspiration [82]. Nevertheless, this approach does not solve the problem of the lack of knowledge of the structure of the embolic material prior to mechanical thrombectomy.

In the literature on the subject, attention was paid to the relationship between the appearance of the symptom of the so-called hyperdense middle cerebral artery sign of the brain shown in the CT scan in the acute phase of stroke with the size of Hounsfield units (HU), which are associated with the number of red blood cells and their parameters, i.e., hemoglobin and hematocrit [83]. According to the accepted theory, the presence of hemoglobin determines the weakening of the clot, and the susceptibility to lysis seems to increase the hematocrit level. Thus, red (cardioembolic) thrombi containing erythrocytes and fibrin result in a higher HU count and greater susceptibility to fibrinolytic agents, while the so-called white clots (atherosclerotic) consisting of varying amounts of platelets, atherosclerotic debris, and cellular debris with low numbers of red blood cells, results in lower HU values and greater resistance to fibrinolytic agents. An additional problem that makes it impossible to generalize the assumptions are clots with a heterogeneous histological structure at different lengths, containing extracellular neutrophil traps and microcalcifications only in specific areas of the thrombus, which together causes resistance to mechanical thrombectomy [76], and, at the same time, their structure cannot be determined in advance with the available diagnostic imaging methods [83].

In addition to thrombolytic activity in blood vessels, rt-PA seems to have a neuroprotective effect consisting in the suppression of oxidative stress during reperfusion, which is associated with increasing phosphorylation of 5’adenosine monophosphate-activated protein kinase, increasing glucose uptake in neurons and promoting mitochondrial ATP production [84].

The knowledge of the physical properties of the clot, i.e., its density or permeability, may influence its successful removal with MT [85,86]. In addition to the above-mentioned clot composition and permeability, clot length and volume are also important for successful recanalization and favorable clinical outcomes in AIS [87,88]. The likelihood of clot dissolution increases, the greater the surface area of the clot (in contact with the blood flow) as determined by cirHU (circle HU) units for the range of blood flow around the clot. CirHU is a marker of good collateral and/or residual flow, which helps in the effective rate of reperfusion, deciding on the accuracy of the adopted indicators [89]. This is especially important in those patients who are less prone to recanalization after intravenous thrombolysis in the initial evaluation [90]. Multiphase angioCT is also used to predict the clinical outcome of AIS EVT, which is used as part of the diagnostic standard to assess the existence (or absence) of collateral circulation to the ischemic area of the brain. Collateral circulation is an important prognostic factor for the volume of the so-called penumbra, i.e., tissue that is ischemic but salvageable after successful recanalization [91,92,93,94]. In addition, it turns out that the patency of the arteries may be genetically determined; in the case of patients with a lower family burden, a greater flow is observed at cerebral arteries [95].

## 4. The Role of Mitochondria in the Pathophysiology of Ischemic Stroke and Recanalization

Cellular mechanisms developing in neurons after reperfusion are still debated. The recanalization therapies in ischemic stroke, apart from their undoubtedly beneficial influence on the restoration of normal cerebral circulation, might also start cellular pathways causing ischemic-reperfusion neuronal death along with changes in the morphology and function of mitochondria with hemorrhage transformation of the tissue necrosis zone after ischemia [96] and the comprehension of these mechanisms may even more improve a patient’s outcome. Impairment of mitochondrial function results from calcium flowing into the cell, which increases its concentration in the mitochondria as early as 24 h after reperfusion procedures [97]. Intra-mitochondrial Ca^2+^ ingress in excess of the buffer capacity leads to osmotic edema of the mitochondrial crest, release of NADH, cytochrome c and cytosolic double-stranded DNA (dsDNA) into the cytoplasm of astrocytes. This, in turn, triggers a cascade of events that lead to increased expression of various pro- and anti-apoptotic proteins by initiating inflammatory responses, GMP-AMP synthase (cGAMP, cGAS), sequentially to cell apoptosis in the penumbra [98,99].

The cycle of events entails the opening of large conduction channels in the inner mitochondrial membrane, known as the mitochondrial permeability transitional pores (MPTP). This, in turn, contributes to the unrestricted penetration of small solutes, disturbance of ion gradients and alteration of the mitochondrial membrane potential, what leads to impairment of oxidative–antioxidant pathways [100]. The activity of proteins related to glycolysis, pyruvate dehydrogenase complex, tricarboxylic acid cycle (TCA), and oxidative phosphorylation are inhibited, which cause metabolic imbalance between the cytosol and mitochondria and lead to breakdowns of energy metabolism [101]. One of the key molecular mechanisms of stroke is believed to be oxidative stress caused by the overproduction of reactive oxygen species (ROS) in the tissues of the brain after the restoration of blood flow or reperfusion. Reactive oxygen species and reactive nitrogen species (including neurotoxic NO) oxidize mitochondrial lipids, protein sulfhydryl groups, and iron–sulfur complexes essential for maintaining the function of respiratory oxidative phosphorylation enzymes. In the case of excess of NO, apoptotic cell death is initiated by activating the p53-dependent pathway, caspase activation, chromatin condensation, and DNA fragmentation [102] As a result of these events, ATP synthesis is inhibited and the p53-dependent mechanism is induced, which is caused by the reaction of NO with O_2_^−^ creating a much stronger oxidant ONOO^−^, which is highly reactive and is mainly responsible for the toxicity of NO in hypoxic brain tissues [103].

In the literature, the oxidative stress induced by damage to the brain tissue was indirectly related to the markers and characteristic metabolic abnormalities, however, the actual dynamics of ROS has not been recorded in vivo so far.

**Figure 1:**
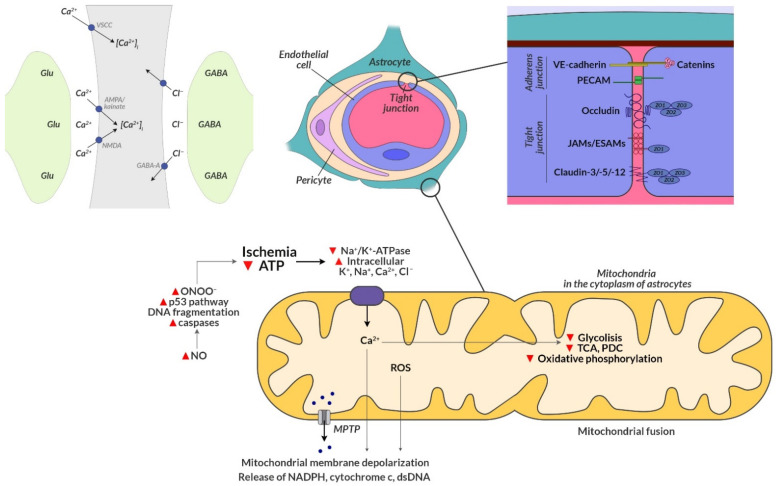
The response of astrocytic mitochondria to hypoxia in the course of ischemic stroke. AMPA: α-amino-3-hydroxy-5-methyl-4-isoxazole propionate; dsDNA: double-stranded DNA; ESAM: endothelial cell-selective adhesion molecule; GABA: γ-aminobutyric acid; Glu: glutamate; JAM: junctional adhesion molecule; MPTP: mitochondrial permeability transitional pore; NADPH: nicotinamide adenine dinucleotide phosphate; NMDA: N-methyl-d-aspartate; NO: nitric oxide; PDC: pyruvate dehydrogenase complex; PECAM: platelet-endothelial cell adhesion molecule; ROS: reactive oxygen species; TCA: tricarboxylic acid; VE: vascular epithelium; and VSCC: L-type voltage-sensitive calcium channels, calcium influx, and overload upon hypoxia.

The resulting oxidative and nitrosative stress induce iron-dependent ferroptosis, a non-apoptotic form of cell death consequently leading to mitochondrial contraction and switching their functions off in the area of neurovascular units (Figure 1) [104,105]. In the course of revascularization procedures, the concentration of cytosolic Ca^2+^ increases secondary to glutamate excitotoxicity, which further inactivates contracted astrocyte mitochondria located near glutamate [106], leading to the death of neurons. Reperfusion is essential for saving ischemic tissue, but, paradoxically, it can also exacerbate neuronal damage by generating mitochondrial damage. Therefore, the timely elimination of dysfunctional mitochondria is crucial for maintaining a healthy mitochondrial network during revascularization procedures [107].

Research is currently under way to precisely define the role of mitochondria in response to the functioning of neurons, astrocytes, and glial cells in ischemic brain damage [100]. The diverse functions of mitochondria in response to ischemia affect all nerve cells, including cortical astrocytes, and are observed with respect to the delivery of glucose-derived ATP energy, mitochondrial membrane potential, cytosolic calcium release, and generation of mitochondrial permeability transitional pore opening (MPTP) in response to pro-apoptotic factors and levels of ketoglutarate dehydrogenase. All the indicated mechanisms related to mitochondrial bioenergetics determine the resistance of astrocytes to ischemia, as well as control the local processes responsible for the survival and death of nerve cells [108,109].

The role of mitochondria in astrocytes is now extensively studied, gaining importance in the emerging field of astroneurology, where small astrocytic mitochondria exhibit neuro-adaptive abilities in an oxygen- and glucose-deprived environment. Thus, astrocyte mitochondria guarantee the maintenance of balance by phagocytosing synapses, fragments of axonal and neuronal mitochondria, and damaged proteins [110]. During hypoxia and lack of glucose, such as ischemic stroke or reperfusion, astrocyte mitochondria are depolarized early, switching glycolysis-related oxygen metabolism to lactate energy supply (a mechanism known as lactate transfer from astrocytes to neurons, astrocyte–neuron lactate shuttle ANLS, associated with simultaneous lactate import and export) [111], released into the extracellular space and taken up by neurons via monocarboxylic acid transporters (MCT), thus preventing neuronal death [112].

Glutamate excitotoxicity related to, inter alia, changes in transport activity as well as altered expression of glutamate transporters, plays a key role in this process, as research shows that calcium activation of glutamate transport by astrocytes seems to be a causative factor affecting the inhibition of mitochondrial function [113,114]. Microglial activation occurs first after cerebral ischemia, and is activated by the M1 and M2 pathways dependent on angiogenic functions and enhancing BBB integrity. This is mainly due to increase of the expression of tight junction proteins (TJP) and the release of matrix metalloproteinases (MMPs) within the neurovascular units (NVU) [115] by inhibiting their functioning and, thus, leading to a metabolic disconnection between the neurons and the proximal blood flow. The change in the architecture of the mitochondria-forming networks is related to the adaptive role of microglia, which determines the survival of surrounding neurons, limiting the extent of losses caused by ischemia-reperfusion damage to the brain [116].

Thanks to these abilities, it is possible to regenerate these organelles and survive in conditions of exposure to environmental stress of ischemic areas of the brain, which is a promising target of therapy improving the clinical condition of patients after stroke and reperfusion procedures.

## 5. The Role of Inflammation in the Pathophysiology of Ischemic Stroke and in the Assessment of the Prognosis of Reperfusion Procedures

Currently conducted clinical trials and animal models indicate that stroke is a network of interactions within the central nervous system (CNS) [117]. In the course of these events, the brain calls for help by releasing a number of factors, such as hypoxia-induced factor 1a (HIF-1a), protein S100b, ATP, which activate the central and peripheral immune systems. By mobilization of the cells of the immune system, i.e., white blood cells, neutrophils, connective tissue cells (mast cells), microglia cells, to the ischemic site, toxic substances of their metabolism are released and involved in the inflammatory response, i.e., pro-inflammatory cytokines, and a number of others, including reactive species of oxygen, nitrogen, sulfur (ROS, RNS), or matrix metalloproteinases, for example MMP-9 [118]. MMP-9 plays an important role in rt-PA-related bleeding complications, while ROS increase the effect of rt-PA on MMP activation in the mechanism of loss of caveolin-1 (cav-1), a protein encoded in the *cav-1* gene, which is a critical determinant of unsealing BBB during reperfusion [119]. Due to the release of toxic components of the blood plasma, apart from the unsealing of the BBB, the blood flow in the area of the cerebral microcirculation is stopped. As one of the released factors of the immune system, cytokines simultaneously stimulate a cellular and/or humoral response. Cells of the peripheral immune system, i.e., monocytes, neutrophils, T-lymphocytes, platelets, and macrophages, get through the damaged BBB from the systemic circulation to the area of cerebral ischemia, contributing to further irreversible ischemic brain damage by penetrating the brain parenchyma and exacerbating the ongoing the inflammatory process [120]. Calprotectin (a heterodimer composed of two cytosolic proteins: S100A8 and S100A9) released by these cells is one of the main proteins in the course of inflammation in patients after ischemic stroke and is a potential predictor of resistance to reperfusion and, thus, determines the effectiveness of reperfusion treatments [121]. In the case of neutrophils, the matter is complicated by their diverse phenotype influencing changes in the structure of these cells depending on the degree of maturity (or related to the presence of TLR4) and differentiation under the influence of regulatory signals released under ischemia [122,123].

The assessment of the amount of released neutrophils after a stroke is a prognostic factor for the severity of a stroke or the degree of bleeding complications. The assessment of an increased neutrophil/lymphocyte ratio is associated with poor neurological improvement after ischemic stroke, which is attributed to the neurotoxic role of neutrophils in the post-ischemic brain region [124].

In general, clinical, imaging, and laboratory biomarkers are important in the assessment of the prognosis of AIS or reperfusion procedures. In predicting annual mortality after ischemia, a worse prognosis is for those with higher NIHSS values and higher carotid intimal and middle membrane thickness (cIMT), lower coagulation parameters, i.e., antithrombin levels, lower platelet count, protein C, and albumin concentration, lower HDL cholesterol, higher concentration of factor VIII, von Willebrand factor (vWF), higher absolute white blood cell count, higher concentrations of tumor necrosis factor α (TNF-α), interleukin 10, high sensitivity C-reactive protein (hsCRP), vascular cell adhesion molecule 1 (VCAM-1), apoB, LDL cholesterol, and triglycerides [125,126]. In the construction of ROC curves of complications and annual survival after ischemic stroke or applied revascularization, a multimodal approach is used that combines a number of biomarkers, i.e., variables using NIHSS, cIMT, age, IL-6, TNF-α, hsCRP, HDL, protein C, protein S, vWF, and platelet endothelial cell adhesion molecule 1 (PECAM-1), which have a larger area under the curve (AUC/ROC), i.e., 0.975 (accuracy ca. 93%, 100% sensitivity, and 85.7% specificity) than either indicators separately [127]. The assessment of monocyte-to-cholesterol high-density lipoprotein (MHR) and monocyte-to-lymphocyte (MLR) ratios as a combined approach is also a better predictor of ischemic stroke/reperfusion than analyzing these parameters separately.

The severity of inflammation associated with damage to the BBB after reperfusion procedures is associated with the risk of hemorrhagic transformation after ischemic stroke [128], being a key factor in its pathophysiology [129]. It is the mitochondria that regulate a number of cellular mechanisms, such as autophagy, apoptosis, energy production, and expression of genes related to mitochondrial biogenesis, thus influencing the direct inflammatory response [108,130] of neurons and, indirectly, of microglial cells [131].

The brain and the immune system constitute functional neuro–humoral connectivity, therefore, the death of nerve cells in the course of acute stroke leads to the release of a number of humoral response factors causing local inflammation in the damaged brain [132]. These factors lead to the expression of receptors on microglial cells and astrocytes, which, in turn, recruit cells of the peripheral immune system to the infarcted brain tissue, consequently contributing to exacerbation of neurological changes. Recruited T lymphocytes and natural killer (NK) cells mediate the impairment of cerebral microcirculation through the adhesion of leukocytes to the cerebral vessel walls and the initiation of secondary micro-thrombosis. Inflammation and/or infection may promote autoimmune responses against brain antigens in stroke patients [133]. Hypoglycemia and insufficient energy to maintain the membrane potential of the nerve cells may degrade the functional assessment of ischemic areas of the brain due to a diminished hormonal response and greater systemic effects [134] independent of the humoral response. The role of the vascular endothelium is crucial in maintaining perfusion and patency of cerebral microcirculation vessels. Brain ischemia causes endothelial damage and a cascade of positive feedback loop events between blood flow and brain tissue along with damage to the BBB, transcytosis, death of endothelial cells, and recruitment of immune cells [135,136].

## 6. Examples of Neuroprotection in the Treatment of Ischemic Stroke

A particular interesting field about acute stroke management is related to neuroprotective therapies. Some reports suggest a potential role of these therapies [137], although underlying mechanisms are still unknown and evidence of their usefulness in clinical setting is lacking [47]. An example of endogenous neuroprotection of damaged neurons after a stroke stimulating the remodeling of the mitochondrial network architecture are the steps of gradual cooling down to 33 °C, which facilitates the transfer of mitochondria from astrocytes to damaged neurons and endothelial cells, while increasing the content of intracellular ATP, mitochondrial membrane potential (MMP), and cell viability during hypoxia [138,139]. Depolarization of the mitochondrial membrane potential of primary cortical neurons is inhibited after excitotoxic glutamate stimulation, which results in NAD^+^ administration, ultimately inhibiting apoptotic neuronal death [140].

Hopes related to neuroprotection are also created by hyperbaric oxygen (HBO) therapy, whose mechanism is associated with the inhibition of mitochondrial apoptosis and disturbances in energy metabolism of these structures [141]. The administration of 100% oxygen at approximately three times higher atmospheric pressure allows for higher arterial oxygen pressure and greater oxygen supply by increasing dissolved oxygen in the plasma, which stimulates cellular respiration and supports ATP synthesis in ischemic areas of the brain [142]. Oxidative stress and the expression of proteins related to apoptosis is alleviated; while the decrease in ATP levels, the activity of enzymes of the mitochondrial complex and the activity of Na^+^/K^+^-ATPase, which maintain the energy metabolism at a constant level, are inhibited [143].

The excessive release of glutamate has become the target of a therapy using checkpoints related to protein kinases regulating the signaling pathways of the N-methyl-D-aspartate (NMDA) receptor [144]. These include mainly antagonists of the glutamate-binding site to the NMDA receptor, e.g., amino acids and their derivatives containing basic acid and phosphate groups with strong hydrophilicity, which makes most of these compounds highly polar and prevents their penetration through the BBB [145]. An equally promising strategy influencing glutamate excitotoxicity is the inhibition of microRNA-29b by suppressing oxidative stress and apoptosis [146]. MicroRNA (miRNA) as single-stranded, relatively short RNA molecules regulate the expression of proteins, thus influencing both physiological processes and diseases of the nervous system. Stroke-specific miRNAs include, inter alia, miR-223, miR-181, miR-125a, miR-125b, miR-1000, miR-132, and miR-124a, which affect glutamate receptors and the related regulation of neuronal and astrocytic proteins after stroke [147,148,149].

Preventing neuronal death and, thus, reducing neurological damage are complex tasks that cannot be successfully solved by targeting individual mechanisms.

One of the treatment strategies discussed in the literature would be the inhibition of TAK1 (transforming growth factor-β-activated kinase 1) in microglial cells in order to protect before stroke by inhibiting the death of neurons, e.g., through the pro-apoptotic JNK/c-Jun pathway. As demonstrated in the mouse MCAO/reperfusion model, TAK1 does not show any neuroprotective activity. Moreover, TAK1 activation was observed in post-ischemic neurons, linking its presence with the death of neuronal cells through many signaling pathways, e.g., JNK/c-Jun, p38 and NF-κB [150]. To date, the molecular mechanism underlying the pathological effects of TAK1 activation in the post-ischemic brain remains unclear. TAK1 inhibition, which promotes gray and white matter integrity, may be a promising therapeutic strategy after ischemic stroke [151].

Post-stroke regenerative therapies currently focus on improving neural plasticity to reverse the disturbances in the nervous structure and improve the functioning of the neural network both during recovery and in the long term.

Stem cell-based therapies are of interest because of their neuro-regenerative potential to promote neurogenesis and protect surviving neurons. Bone marrow stem cells (BMSC) and mesenchymal stem cells (MSC) promote neurogenesis in preclinical models of intracerebral transplantation with immunomodulatory properties capable of suppressing inflammation following stroke and, potentially, improving recovery [152].

Cell therapies based on protective cell phenotypes are also an interesting approach of improvement after reperfusion therapy in ischemic stroke patients. Polarized microglial cells or peripheral blood mononuclear cells are promising therapeutic strategies because of their pleiotropic effects, which, depending on the microenvironment and adaptation properties, determine the variable phenotypes of cells in response to brain injuries [153,154]. Microglial activation has significant effects on spontaneous regeneration after stroke, including structural and functional restoration of neurovascular networks, neurogenesis, axonal remodeling, and blood vessel regeneration. Polarized cell therapies are gaining increasing attention in the treatment of strokes and neurological diseases [155].

An enhanced immune system response during ischemic stroke is the target of advanced immunotherapies of ischemic inflammatory cascade while extending the therapeutic time window [156] over conventional rt-PA treatment or revascularization therapy. An example is natalizumab, a monoclonal antibody that shows therapeutic effects in both preclinical and clinical trials related to stroke [157]. The mechanism of action of the used biological therapy is that the antibody interacts with its specific target, be it a ligand or a receptor, inhibiting the cytotoxic signaling cascade. Thus, immunotherapy improves cell viability or delays cell death [158], therefore ultimately reducing inflammatory injuries and, at the same time, stimulating peripheral immunity by acting neuroprotectively. Natalizumab prevents systemic migration of brain leukocytes or inhibits the neurotoxic production of inflammatory mediators, excluding the incidence of bacterial infections during stroke changes. It should be noted, however, that stem cell therapies bring better results mainly among people who are prone to stroke.

Targeting receptors or proteins of channels and enzymes (CD68, Iba-1, GFAP) involved in microglia-dependent neuritis is a promising goal of therapy after recanalization procedures, especially in the treatment of secondary damage caused by cerebral ischemia (where many types of cells are involved, including microglia, astrocytes, oligodendrocytes, and peripheral T lymphocytes) [159]. This is due to a different phenotype of neurodegenerative lesions resulting from secondary thalamic trauma compared to the primary cortical injury following stroke. Signaling changes from initiated nervous system inflammation appear to represent a key canonical signaling pathway between primary and secondary changes in both brain regions following stroke [160].

## 7. Potential Goals of Ischemic Stroke Therapy and the Evaluation of the Effectiveness of the Applied Treatment

Selection of endpoints is a significant problem in assessing the effectiveness of the applied recanalization procedures both in clinical and pre-clinical evaluation. This applies in particular to the confirmed scientific evidence on the effectiveness of new drugs, which would be classified in terms of not only survival or functional improvement expressed according to the modified Rankin Scale (mRS) as the main endpoint, but also other measurable scales for the obtained results, e.g., independent measurements of imaging or laboratory tests [161]. Examples include the determination of the perfusion volume, the volume of the ischemic intact part of the brain, or real-time histomorphometric measurements of the embolic material during reperfusion procedures.

The search for a possible treatment of ischemic stroke has evolved from finding a relationship between selective molecular events controlling the known mechanism of action of selected individual stages of the ischemic cascade, such as the use of glutamate receptor-specific antagonists (for which clinical trials have failed) to synergistic and pleiotropic therapies combining glutamate antagonists and GABA agonists [162], which provided scientific evidence of their effectiveness. Similarly, the consideration of single peripheral markers for assessing recanalization efficacy and long-term functional benefit after stroke was more predictive of the concept of combining multiple biochemical biomarkers than considering each of the indicators individually.

Based on our previous experience related to the search for objective biomarkers of schizophrenia, which is characterized by the complexity of clinical symptoms and endophenotypic differentiation, we can draw conclusions that one must look at a new perspective in the search for the prognosis and treatment of strokes related to the cascade of events [163]. In the constructed statistical model, the results of clinical, imaging, and laboratory tests will be taken into account in a number of variables, which will allow to achieve long-term success only when taken together.

The ongoing efforts focused on a number of variables covering all cells of the nervous system, i.e., neurons, astrocytes, and cells that form the blood–brain barrier, are focused around translational medicine. From the laboratory table and in vitro research, it will take the changes at the level of preclinical and clinical research involving the human brain, setting a new horizon of thought presumably capable of predicting complex neurological functions.

A limitation of this approach is the complex nature of the neurovascular units (NVU) that form networks of connections throughout the brain that cannot be linked to unicellular in vitro models. Studies show the isolated functions of individual types of NVU cells, ultimately causing many problems of an interpretative nature in relation to the functions performed in vivo [164,165], in particular in the area of the ischemic penumbra, where the observed residual blood flow supporting cell survival cannot be reproduced in vitro.

Single-cell models, despite a number of limitations in relation to in vivo models, are simple and repeatable, thus, it becomes possible to control potential candidates for neuroprotective drugs and to assess their protective effects against glial cells and the functioning of the blood–brain barrier.

Studies on the circulation of mitochondria between astrocytes and neurons during ischemia/reperfusion provide evidence of potential new targets for stroke therapy to address mitochondrial-related energy failure. Initial preclinical studies related to the rescue of neurons through astrocytic mitochondria have shown the first spectacular results in this area [166].

Despite the triumphant recanalization therapies, thrombolysis and thrombectomy in the treatment of ischemic stroke, currently perceived treatment will require a redefinition of basic concepts to recognize the differential susceptibility associated with the complexity of the neurovascular units that make up the brain networks, requiring a pleiotropic approach to work through many different mechanisms of action.
